# Plates and Antagonizers

**Published:** 1857-06

**Authors:** H. R. Smith


### PLATES AND ANTAGONIZERS.

BY H. R. SMITH.

My last article was devoted to preparing models and metallic casts. In this I proceed to aid the student on in his operations of making a full denture of artificial teeth. And as the time for spiral springs is nearly gone by, I shall at this time only describe the manner of fitting by atmospheric pressure or suction plates. For by a proper care in manipulating, very few cases will present themselves to us but what can be done without springs.

There are some cases, however, where there is some malformation of the mouth, or irregularity caused by disease, when springs are indispensable. Such cases will be spoken of in some future article.

Our metallic casts being ready, we first get a pattern plate of sheet lead, which should be a little thicker than our gold plate, or about No. 25 or 26 of our gauge plate.

The patterns should be well trimmed and fitted to the casts, which being done, we should, if possible, try the lead plates in the mouth of the patient. If not trimmed sufficiently, it should now be done, until they rest free and at ease in every part; and if these lead plates fit the mouth accurately, we can proceed with our work, with full confidence that with care our gold plates can be made to do the same. But if they do not, of course the casts would not make the gold plate sit any better.

By trying our lead plates, any peculiarity of the mouth and gums will generally be noticed, so that we can, if we wish, make any alteration which the case may require. Some might object to this, for the reason that it puts us to some extra time and trouble. But as success is what we aim at, we should not consider our time misspent if it adds to our success.

Our work so far being satisfactory, we now flatten out our patterns, and by them cut our gold plate. For an upper plate, the gold should not be thinner than No. 27 or 28 gauge, while the under should be as thick as 23 or 24, if a single plate is used, or what is better, made double from the same thickness of plate as the upper. The double plate has greater strength and elasticity than the same thickness of single plate.

For fitting the plates to the cast, wooden or horn mallets should be used, for hammers jam and harden the gold, making the plates thinner in some places than in others, and such places, when finished up, are liable to be too thin, also liable to be melted through while soldering, if our solder is as fine as it should be. And again, plates of unequal thickness are more liable to spring while soldering, as those places will be more heated than the balance of the plate. During the fitting, the plate should be annealed very often, to prevent it from cracking.

After fitting and gauging with the first set of casts, then gauge with the second, and fully define the air chamber. The under plate may be fitted in one piece, or in two. If in two, the ends at the median line should be passed by each other to a short distance, and soldered. Then, if a second plate is used to double the first, it should so be put on that the joints should not come in the same place. After the plates are properly fitted to the casts, anneal them well and evenly, taking care to only bring the plates to a dull red, for if the heat is raised too high, the texture of the gold will be destroyed, and made more liable to spring in soldering.

The plates should now be cleaned in the acid bath, and well washed, and they are ready to try in the mouth. If when tried in the mouth, the plates need more trimming, it should be done until they sit perfectly easy and free from all undue pressure or cutting.

Now take a small vial cork, and place between the plates in the mouth, to determine the distance you want the plates apart. If too long, trim the cork until the mouth rests in an easy and natural position. This will determine the length of the teeth when the mouth is closed. Now cut a strip of plate tin or zinc the width that the cork is long between the plates, three inches long. This is your guide.

Put the under plate into its position in the mouth, warm wax, as for an impression, and put on to the upper plate about three-fourths of an inch thick. Run the guide through the wax with one edge next the plate at the median line. Now place the plate and wax in the mouth, and direct the patient to close the mouth carefully, until with the under plate he hits the under edge of the guide. Hold the mouth in this position, and with the finger press the wax close to the plates, all round the labial surface.

Now remove the whole, plates and wax together, let the wax cool so as not to be displaced, heat the end of the guide in the spirit lamp until it can be easily withdrawn, and remove it carefully. Now trim the wax on the labial surface, to represent the fullness you desire your teeth to present when they are on the plate. Try the plates with the wax in the mouth, to determine whether the distance and fullness suits you, also whether the patient closes his mouth naturally.

If you have your teeth at hand, it is well, at this stage of the operation, to select the front ones, and place them on your wax, and try them in the mouth. By so doing, you can better judge as to color suitable for the case, also gratify the peculiar fancy of your patient in that respect, and perhaps save a dissatisfaction which might possibly accrue afterwards. For as far as the operator can go without doing his skill injustice, so far is it the patient's right to have his taste gratified.

Now remove the plates and wax from the mouth, take off the teeth, and you are ready to make your antagonizers. Give the plates and wax a coat of oil with a camel's hair pencil, then surround it with a strip of sheet tin, three inches in width, letting it extend one and one-half inches beyond the heel of the plates, and turned so as to make the ends meet to make a box.

Turn on plaster to the depth of one inch above the plate, and when it is hardened sufficiently to handle, take it out of the tin box, turn it over, and cut some notches each way across the projecting portion at the heel, so that when the other side is on, it will not turn and change its position while grinding in the teeth.

Varnish over the portion that will come in contact with the other side, and then give it a coat of oil, to keep the other side from adhering when turned on. The varnish keeps the oil from striking into the plaster. Now surround again with the tin box, and pour plaster on the other side, and when hardened, trimmed and varnished, you have your antogonizers, or artificial jaws, with the plates in the same relative position as they had in the mouth.

Many antagonizers have been brought out before the profession, some of which are very complicated, and all claiming much merit, but to my mind, none of them equals, taking into consideration all things, the plaster antagonizers.

If due care is taken in carrying out the above directions, the operator will rarely fail in getting a correct antagonism. And next to a correct impression, is a correct antagonism; for if you fail in the last, even if the first is good, a bad job will be the result.

If the operator has no regard in saving the natural grinding surface perfect, he can antagonize his teeth after they are soldered on to his plates, by grinding them to fit each other. But if perfection is his aim, he will try to preserve the grinding surface on the teeth as near as possible as they come from the manufacturer, which will make perfect antagonizers indispensable.

Now separate the wax equi-distant from each plate, and you are ready to grind on the teeth. No exact rule can be given for selecting the teeth, as that will depend upon the peculiar circumstances attending each case. The age and complexion of the patient, size and peculiarities of the jaws, will be, for the most part, the governing indications, and must be well studied by the operator who would be a true artist in his profession.

If there are under teeth remaining, and only an upper set is wanted, the same directions will be followed until we come to selecting teeth and grinding them in. Then the remaining teeth will govern the color and position in which they are to be set. In grinding in the teeth, much taste can be exercised in making them look natural and easy, so as to avoid detection. When the teeth are ground on, they should have as equal a bearing on the plate as possible; for if they do not, they will be liable to spring the backings, and soon break off.

There is one difficulty which many operators meet with, which is, that after the teeth are soldered on, they find them spring off from the plate at the heels. This is owing to the expansion of the teeth when heated up to solder, and while they are expanded, they are drawn back from the plate, and soldered in that position, and therefore they can not go back to the plate when cold.

I will now describe the method which I pursue, and which I think will in every case remedy the evil. It is this: Grind on the teeth as usual, fitting them just as you want them to stand when the job is done, and fasten them well by heating the wax around the pivots of the teeth, with a small instrument heated and passed down the inside of each tooth.

Then take each tooth off one by one, and grind off a little from each approximal surface of the gum, and then put the tooth back, and fasten it where it was before; and so remove each tooth separately, and replace it until all are done. Then there will exist a perfect space between all of the teeth, to give room for expansion without throwing the teeth off from the plate while soldering. And when the teeth are soldered on, they will not only fit close to the plate, but also close to each other.

Another difficulty is experienced by operators, which is springing of their plates while soldering. So great has this difficulty been, that very many able articles have been constantly appearing in our journals on the subject, until the Editors asked us if we could not give it some other name, for they were tired of heading articles in every issue, "Springing of Plates." But with all the plans proposed and certain remedies promised, the difficulties still exist. Now what shall we do? Because the subject is worn thread-bare, shall we cease to mind and talk about it? Is it not even more necessary than to talk of the thousand and one subjects which meet a dentist only once in a life-time?

I think that operators should not relax their efforts to find out and remedy the cause, if possible, and at the same time inform the profession of their proximate success. It is the every-day difficulties which are of the most importance to the operator, and to such let those who know where the remedy is, devote their pen. Let your lights shine.

There are several causes which operate to spring our plates, some of which are, binding of the teeth too close, where they must expand, the material for investing, manner in which they are invested, backings badly fitted to the plate and to each other, heating up too suddenly and unevenly, and poor solder.

After I have soldered a job, I save the investing, and grind it up and put in a drawer for use. When I am ready for investing my job, I prepare my investing of new plaster, two parts, asbestos one part, and the old material one part, mixed up to the consistency that I wish. This material does not shrink so much, or crack when heated up, dries much quicker than plaster and sand, and cools much more evenly.

For both upper and under jobs, I prepare a copper wire of one-eighth inch size, and bend it so as to entirely surround my job at about one-eighth of an inch away from the teeth, letting it go around the heel, to lock with hooks bent on the ends. This wire I imbed in the investing around the job, about midway of the length of the teeth, so that it shall not touch the teeth or plate at any point. And for the upper job I use, in addition, an iron wire the size of the copper, bent to fit the furrow for the alveola in the plate. This wire I imbed in the investing in the furrow, so that it shall not touch the plate, and let it extend one-fourth of an inch beyond the heel of the plate. Then cover the whole one-half inch thick with the investing. Let the whole dry, and then back the teeth as usual, either by removing, or remaining in the investing. I use thick backings, No. 24, and let them join as far as the gums come up on the teeth, then use solder as fine as the plate will permit. Borax the backings well with borax ground in water before heating up, heat up slowly and evenly in a charcoal or coke fire, put on the solder, and flow quickly. Then cool off slowly, remove the investing, and pickle ready for dressing up.

The iron wire prevents too much lateral shrinkage, and the copper wire prevents cracking of the investing, and counteracts, in a great measure, the expansion of the iron wire.

With the above directions well observed, there is but little liability of injuring the teeth, or springing the plates in soldering.

